# A population data-driven approach to identifying ‘Long COVID’ cases in support of diagnosis and treatment.

**DOI:** 10.23889/ijpds.v7i3.1924

**Published:** 2022-08-25

**Authors:** Jennifer Enns, Alan Katz, Marina Yogendran, Marcelo Urquia, Saman Muthukumarana, Surani Matharaarachchi, Alexander Singer, Nathan Nickel, Leona Star, Teresa Cavett, Yoav Keynan, Lisa Lix, Diana Sanchez-Ramirez

**Affiliations:** 1 University of British Columbia; 2 First Nations Health and Social Secretariat of Manitoba

## Objectives

Post-acute COVID-19 (or ‘long COVID’) manifests as a wide range of long-lasting symptoms affecting multiple organ systems. We are developing criteria for identifying long COVID cases using administrative, clinical, survey and other data from Manitoba, Canada, with the ultimate goal of examining long COVID prevalence, risk factors, prognosis and recovery.

## Approach

Given the lack of an accepted clinical definition and resulting lack of diagnostic codes, we are adopting several different creative and complementary strategies to identify long COVID cases. We are examining administrative and clinical data sources (laboratory data, physician claims, drug prescriptions, and electronic medical records) for information on positive COVID tests, common symptoms and complaints, and treatment provided. To identify people with long COVID who may not have sought healthcare, we are collecting survey data from a convenience community sample (members of a medical health fitness facility) and mining data on long COVID symptoms from Twitter.

## Results

The combination of approaches we have adopted and the expanding scientific literature on long COVID are contributing to a more comprehensive understanding of the impacts of long COVID in Manitoba. Through preliminary work on the laboratory data (positive COVID tests March 2020-June 2021), we have developed and characterized a COVID-positive cohort (n=47,515). Work is now underway to develop an algorithm for long COVID using symptoms from free text in electronic medical records, ICD-9 codes, and changes in health-seeking behaviour (compared to the pre-positive COVID test period and a matched sample). This population data-driven approach will then allow us to examine how multiple underlying health conditions, COVID illness severity, COVID vaccination status, and various socio-demographic factors are related to risk of long COVID.

## Conclusion

This research is generating actionable information by identifying risk factors to support clinical diagnosis of long COVID, making it easier for clinicians to recognize this new illness and develop plans to manage it, and will inform healthcare system planning by quantifying the burden of long COVID at the population level.

